# Circulating microRNA associated with future relapse status in major depressive disorder

**DOI:** 10.3389/fpsyt.2022.937360

**Published:** 2022-08-17

**Authors:** Qingqin S. Li, David Galbraith, Randall L. Morrison, Madhukar H. Trivedi, Wayne C. Drevets

**Affiliations:** ^1^Neuroscience Therapeutic Area, Janssen Research and Development, LLC, Titusville, NJ, United States; ^2^JRD Data Science, Janssen Research and Development, LLC, Titusville, NJ, United States; ^3^Rancho Biosciences, San Diego, CA, United States; ^4^Department of Psychiatry, Peter O’Donnell Jr. Brain Institute, UT Southwestern Medical Center, Dallas, TX, United States; ^5^Neuroscience Therapeutic Area, Janssen Research and Development, LLC, San Diego, CA, United States

**Keywords:** circulating miRNA, depression, relapse, *hsa-miR-199b-5p*, *hsa-miR-215-5p*

## Abstract

Major depressive disorder (MDD) is an episodic condition with relapsing and remitting disease course. Elucidating biomarkers that can predict future relapse in individuals responding to an antidepressant treatment holds the potential to identify those patients who are prone to illness recurrence. The current study explored relationships between relapse risk in recurrent MDD and circulating microRNAs (miRNAs) that participate in RNA silencing and post-transcriptional regulation of gene expression. Serum samples were acquired from individuals with a history of recurrent MDD who were followed longitudinally in the observational study, OBSERVEMDD0001 (ClinicalTrials.gov Identifier: NCT02489305). Circulating miRNA data were obtained in 63 participants who relapsed (“relapsers”) and 154 participants who did not relapse (“non-relapsers”) during follow-up. The miRNA was quantified using the ID3EAL™ miRNA Discovery Platform from MiRXES measuring 575 circulating miRNAs using a patented qPCR technology and normalized with a standard curve from spike-in controls in each plate. The association between miRNAs and subsequent relapse was tested using a linear model, adjusting for age, gender, and plate. Four miRNAs were nominally associated with relapse status during the observational follow-up phase with a false discover rate adjusted *p*-value < 0.1. Enrichment analysis of experimentally validated targets revealed 112 significantly enriched pathways, including neurogenesis, response to cytokine, neurotrophin signaling, vascular endothelial growth factor signaling, relaxin signaling, and cellular senescence pathways. These data suggest these miRNAs putatively associated with relapse status may have the potential to regulate genes involved in multiple signaling pathways that have previously been associated with MDD. If shown to be significant in a larger, independent sample, these data may hold potential for developing a miRNA signature to identify patients likely to relapse, allowing for earlier intervention.

## Introduction

Major depressive disorder (MDD) is an episodic condition with a relapsing and remitting disease course that has been linked to altered function and genetic variation in neural circuits and molecular pathways which regulate neuroimmune, neuroendocrine, and behavioral responses to stressors and opportunities for reward (1, 2). It has been reported that clinical features such as rumination, subthreshold inter-episodic depressive symptoms, family history, early MDD onset, and family history of mental illness predict relapse/recurrence (3, 4). The relationships between biological pathways implicated in MDD and episodes of illness relapse or recurrence remain inconclusive. A recent meta-analysis examining the role of cortisol in predicting future depressive episodes with a small effect size, however, sensitivity analysis revealed that the results became insignificant once applying outlier removal and/or exclusion of low-quality studies (5). Elucidating the neurobiological mechanisms which govern transitions in and out of illness episodes in MDD holds importance both for optimizing the clinical management of MDD and for guiding the development of novel treatments that more effectively prevent relapse and recurrence (6). Moreover, the identification of novel peripheral biomarker signatures that predict or correlate with relapse risk in MDD holds the potential to complement clinical assessments in precision medicine approaches to prognostic predictions.

Genetics plays an important role in the disease etiology of MDD and this has been characterized by large-scale consortium and cohort studies (7, 8). Gene-environment interactions appear to play a particularly important role in the pathogenesis of MDD, with early-life stress (ELS) constituting an important risk factor for many psychiatric disorders including MDD (9). ELS exerts its effect via epigenetic regulations and results in sustained hypothalamic-pituitary-adrenal (HPA)-axis dysregulation (10–12). Epigenetic regulations including deoxyribonucleic acid (DNA) methylation, histone modification, and non-coding RNA regulation play an important role in MDD (13–18). microRNA (miRNA) is the most common form of non-coding RNA and binds to the 3’-untranslated region (UTR) of its target mRNA and modulates the mRNA transcription (19). miRNA exists intracellularly but also in bodily fluids such as plasma, serum, and cerebrospinal fluid (20, 21). Extracellular exosome vesicles have been described in bodily fluid and are signaling messages mediating cell-cell communication (22, 23). Circulating RNAs including both the miRNA in extracellular vesicles including exosome vesicles and vesicle-free miRNA can be quantified easily without explicitly isolating the exosome fraction and emerge as a new class of biomarkers (24).

The roles of circulating miRNAs have been reported in other disease conditions such as coronary artery disease (CAD), diabetes, cancer, and autism spectrum disorder (25–28). In MDD, the roles of circulating miRNAs are also being characterized. Circulating *hsa-let-7e-5p, hsa-miR-125a-5p, miR-34b-5p, miR-34c-5p, miR-451a, miR-17-5p*, and *miR-223-3p* were up-regulated in plasma samples from patients with MDD and/or bipolar disorders compared to healthy controls (29–31), while *miR-320a, miR-134* and *miR-144-5p* were down-regulated (29, 32, 33). The downregulation of miR-134 was also observed in the chronic unpredictable mild stress (CUMS) rat model of depression (33). The plasma miR-144-5p expression level was inversely related to the Montgomery-Asberg Depression Rating Scale (MADRS) depression score (32). Lower plasma levels of hsa-let-7b-5p are associated with a higher future risk of MDD (within 5 years) (34). let-7b-5p, let-7c-5p, miR-374b, and miR-10a were also downregulated in blood, and miR-508-3p and miR-152 were downregulated in the prefrontal cortex in patients with MDD compared to controls (35–37). Among these miRNAs, miR-34b-5p and miR-34c-5p expression levels were negatively correlated with NOTCH1 (30), while *GRIN2A* and *DISC1* are the predicted targets for miR-320a and *SLC17A7* is the predicted target for miR-451a, miR-17-5p, and miR-223-3p (29). There are quite some miRNAs implicated, and consistent players are yet to be revealed as more studies are conducted allowing for meta-analysis.

In this study, we used an MDD sample to study the relationship between circulating miRNA in serums and the future risk of relapse. Understanding biomarkers predicting future relapse episodes are useful in identifying the subjects with a worse disease course and has prognostic potential.

## Materials and methods

### Study cohort

A total of 217 blood serum samples from patients with MDD were collected from the observational clinical study, OBSERVEMDD0001 (ClinicalTrials.gov Identifier: NCT02489305), in which patients were prospectively followed for a maximum of 2.8 years for relapse detection. The entrance criteria included requirements that participants met the Diagnostic and Statistical Manual of Mental Disorders (DSM)-V criteria for non-psychotic, recurrent MDD, and had experienced the onset of the most recent major depressive episode within the 24 months before screening. In addition, patients must have been receiving an oral antidepressant pharmacotherapy (at an approved therapeutic dose) to which they had recently responded or remitted (within the past 3 months), as documented by a MADRS total score of ≤ 14 at both the screening and baseline visits. The serum samples used for the miRNA assays were drawn from the participants at either a baseline visit or follow-up visit. The mean sample storage duration prior to the assay is presented in Table 1. For both subjects who relapsed and those who did not relapse during the observational follow up phase, the miRNA assay was obtained for only a single time point; for the participants who ultimately relapsed, the biosample used for the assay was drawn before the relapse event (the mean time interval between sampling and relapse declaration appears in Table 1).

**TABLE 1:** Demographic and clinical characteristics of the samples.

	Relapser (*n* = 63)	Non-relapser (*n* = 154)
*N* (%)		
Sex		
Female	41 (65.1)	111 (72.1)
Race		
White	51 (81.0)	127 (82.5)
Black or African American	8 (12.7)	19 (12.3)
Asian	0 (0)	4 (2.6)
American Indian or Alaska Native	1 (1.6)	0 (0)
Multiple	0 (0)	2 (1.3)
Other	1 (1.6)	2 (1.3)
Not Reported/Unknown	2 (3.2)	0 (0)
Mean (SD)		
Age, years	45.59 (12.64)	42.78 (12.96)
Sample was taken before event/censor, weeks	16.9 (11.6)	8.6 (9.1)
Sample age, years	4.73 (0.41)	3.90 (0.69)
MADRS score at the time of blood draw	8.25 (5.17)	5.47 (5.37)

A relapse event was defined by the occurrence of any of the following: (a) MADRS total score greater than or equal to 22 on at least two consecutive visits, with an interval of 1-2 weeks; (b) Hospitalization for worsening of depression; (c) Suicidal ideation with intent or suicidal behavior, and (d) as determined by the clinical investigator. In addition, a single occurrence of MADRS total score ≥ 22 not verified by a follow-up visit within two weeks was also considered a relapse if it was followed by a verification visit (within 23 days) and (i) CGI-S change-from-baseline = 2 or (ii) Medication change suggestive of worsening symptoms (increased dose or augmentation) within 14 days of MADRS = 22 (14-day window preceding or following MADRS = 22). The relapse events were confirmed by adjudication from clinicians.

### Blood collection and serum processing

Patients were instructed to adhere to a low-fat diet on the day of sample collection. A volume of 8.5 ml blood was collected via venipuncture using a gold top serum separation tube (SST). The clinical sites were instructed to thoroughly mix the blood with the clotting activation agent by inverting the tube no less than five times and to allow the blood to clot for at least 30 min. The blood tube then was centrifuged at room temperature [minimum of 1,500 g for 15–20 min] until clot and serum were separated by a well-formed polymer barrier. The serum was aliquoted to four cryovials to minimize the freeze and thaw cycle and frozen.

### Serum microRNA quantitative polymerase chain reaction assay

The absolute expression (copy numbers) of 575 candidate miRNAs were quantified in each patient and control biospecimen using ID3EAL miRNA Discovery Platform using a miRNA-specific RT-qPCR assay (MiRXES, Singapore) via a controlled workflow described in detail previously (38). Total RNA from 200 μl of patient serum specimen was isolated using miRNeasy serum/plasma miRNA isolation kit (Qiagen, Germany). Two sets of synthetic spike-in miRNA controls (three each at high, medium, and low concentration) were added to samples before RNA isolation and before RT-qPCR to monitor and normalize technical variations throughout the entire experiment. The absolute expression of each miRNA was normalized using calibrated spike-in controls and determined for each patient’s serum sample (expressed as log 2 copy number/ml serum). The samples were assayed across two plates in this study.

### Data preprocessing and statistical analysis

For samples below the lowest level of detection (and therefore missing), it was set to a plate-level minimum where feasible. miRNA with greater than 90% missingness were excluded from the statistical analysis.

Although this is an absolute miRNA quantification assay, we used R package variancePartition (v1.20.0) (39) to understand the source of variation in the qPCR experiment. Factors such as age, gender, race, plate, sample age, and relapse status were considered (Supplementary Figure 1). The relapse status was analyzed both in a linear regression framework and in a cox regression survival analysis framework. For the binary endpoint, a linear model was fitted using R package limma (v3.46.0) (40) to identify miRNA predicting relapse status in the observational follow-up phase, while correcting for age, gender, and plate. miRNA associated with relapse status with a false discovery rate of less than 0.1 was reported. For the top miRNA identified, a Cox proportional hazards regression model using R package survival (v3.2-7) (41, 42) was also fitted to leverage the time to event information, and the miRNA level was also categorized as low, medium, and high expressed level for Kaplan–Meier curve plotting using R package survminer (v0.4.9) (43).

### Over-representation analysis of putative targets of differentially expressed microRNAs

Ingenuity Knowledge Base (QIAGEN, Redwood City, CA, United States) is a database with curated and integrated miRNA targets from various sources and literature [miRecords (44), TarBase (45), TargetScan (46), and Ingenuity Expert Findings] and classified the targets into three groups: experimentally observed, predicted with high confidence [cumulative weighted context score (CWCS) less than -0.4 for TargetScan v7.2], and predicted with moderate confidence (CWCS between -0.2 and -0.4 for TargetScan v7.2). Over-representation analysis (ORA) (47) of experimentally observed targets from Ingenuity Knowledge Base and a few manually curated targets were performed using the Kyoto Encyclopedia of Genes and Genomes (KEGG) database (48) and R package clusterProfiler v3.18.1 (49). ORA was performed using additionally performed using the Gene Set Enrichment Analysis (GSEA) resource^1^ which assumes a broader background gene set and test overrepresentation at higher levels of the ontology hierarchy. Gene ontology databases used included c2.cp and c5 subsets of Molecular signatures database (MSigDB) (50).

## Results

The demographic and clinical characteristics of the participant sample are described in Table 1. Of 217 participants, 63 (29.0%) relapsed. The average MADRS score at the time of blood draw for subjects who relapsed during the follow-up phase is 8.25 ± 5.17, and the relapse event occurred 16.9 ± 11.6 weeks later on average. The average MADRS score at the time of blood draw for subjects who were censored (i.e., did not relapse during the follow-up phase) is 5.47 ± 5.37, and the censor occurred 8.6 ± 9.1 weeks later on average.

**FIGURE 1:**
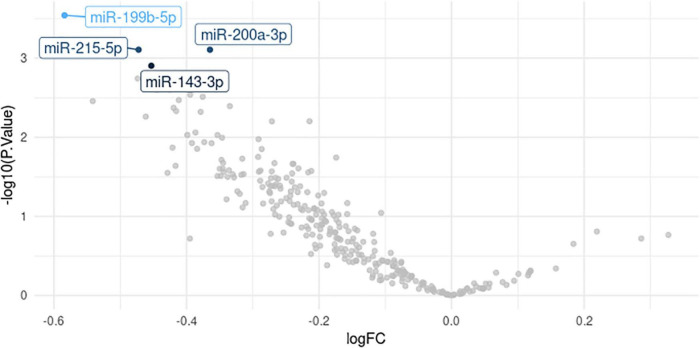
Volcano plot. logFC for relapser vs. non-relapser was plotted against -logP, where *P* is the association *p*-value.

**FIGURE 2:**
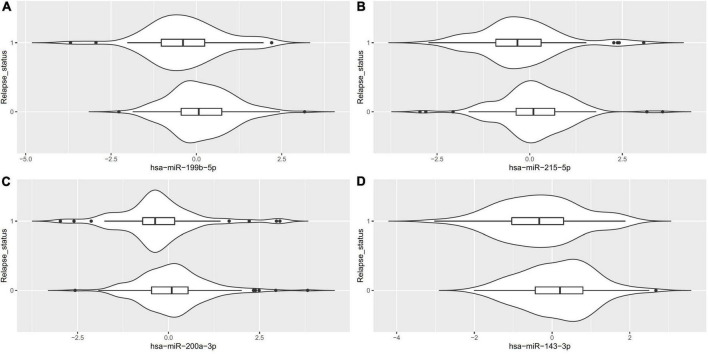
Violin plots for the differentially expressed miRNA (FDR < 0.1) between patients who relapsed during the follow-up phase (1) vs. those who did not (0).

**FIGURE 3:**
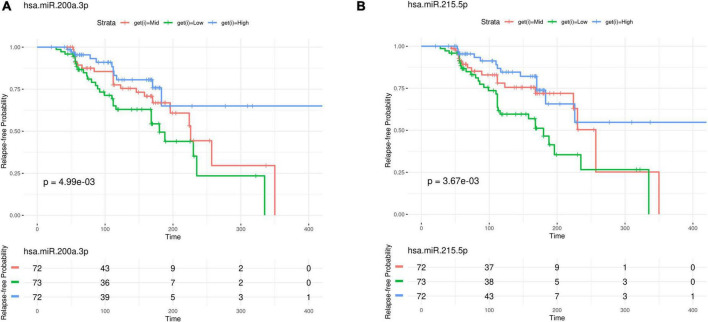
Kaplan–Meier curves for **(A)** *hsa-miR-200a-3p*, **(B)** *hsa-miR-215-5p*.

Of the 575 miRNAs profiled with this technology, 287 miRNAs passing the 90% missingness filtering were included in the statistical analysis. Four miRNAs were nominally associated with relapse status during the follow-up phase (false discovery rate (FDR) adjusted *p*-value < 0.1, Figure 1 [volcano plot] and Table 2). Specifically, lower levels of hsa-miR-199b-5p (Figure 2A and Supplementary Figure S3A), *hsa-miR-215-5p* (Figures 2B, 3B), *hsa-miR-200a-3p* (Figures 2C, 3A), *hsa-miR-143-3p miRNA* (Figure 2D and Supplementary Figure S3B) were associated with risk of future relapse (Table 2). Overall, more miRNAs trended toward being down-regulated than up-regulation in the subgroup who relapsed versus the subgroup who did not relapse. The experiment used a balanced block design (Supplementary Figure 1 and the design table for the study) to minimize technical artifacts. Except a few miRNAs, none of the factors examined explained a significant proportion of the total variability of miRNA expression levels. For the four miRNAs identified, the variance fractions explained by plate or other factors did not dominate the total variance explained (Supplementary Figure 2). For example, for *hsa-miR-199b-5p*, 5% of the variance was explained by the risk of future relapse, while 0.074, 2.73, 3.32, 0.18, and 0.038% of the variances were explained by sex, age, race, sample age, and plate, respectively. Using the Cox proportional hazard regression framework, all four miRNAs were significant with *p* < 0.05 (Supplementary Table 1).

**TABLE 2:** microRNA (miRNA) associated with relapse status with FDR <0.1.

	logFC	AveExpr	*t*	*P*-value	adj.*P*.Val
hsa-miR-199b-5p	-0.58	16.59	-3.69	0.000288	0.07
hsa-miR-215-5p	-0.47	16.19	-3.41	0.000783	0.07
hsa-miR-200a-3p	-0.36	17.03	-3.41	0.000783	0.07
hsa-miR-143-3p	-0.45	21.10	-3.27	0.001249	0.09

**FIGURE 4:**
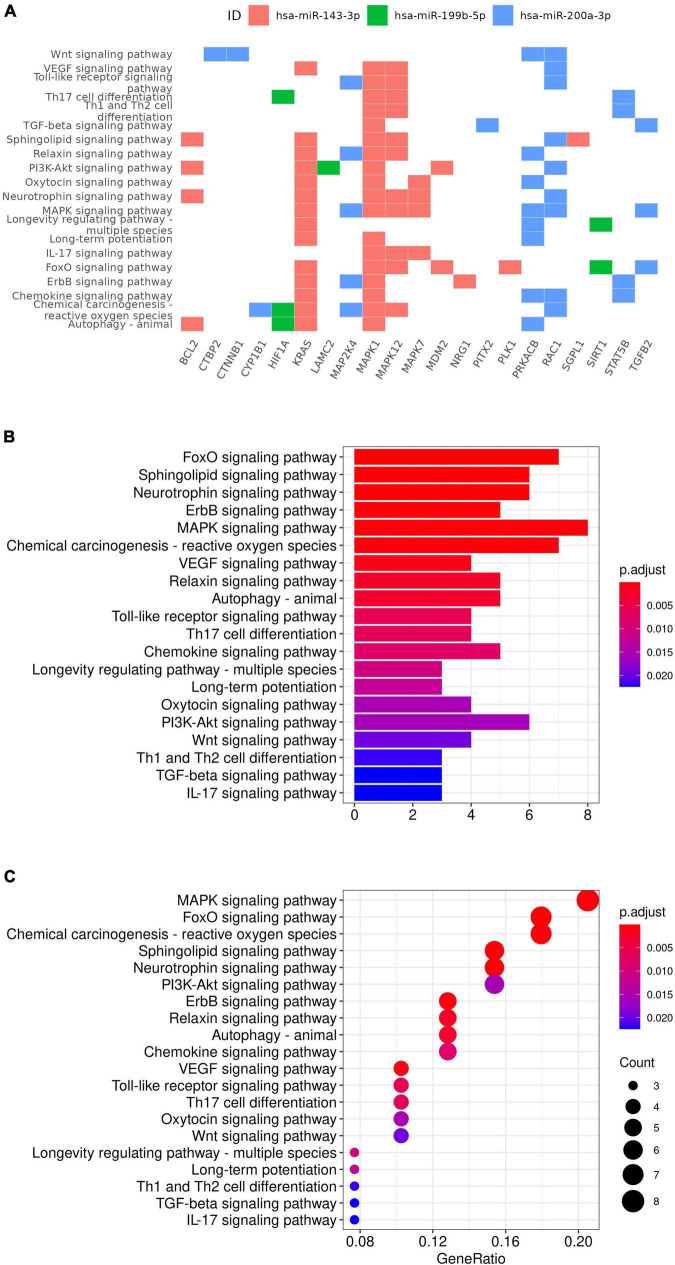
KEGG gene set enrichment analysis results. Targets of miRNA were plotted against the enriched pathway **(A)**. Selected enriched gene sets were also plotted with *p*-value **(B)** and gene ratio **(C)** on x-axis.

Ingenuity Knowledge Base revealed 44 unique experimentally validated targets of the four miRNAs identified and two of them, *ZEB1* and *ZEB2*, were targets for both *hsa-miR-215-5p* and *hsa-miR-200a-3p* (Supplementary Table 2). Enrichment analysis of these experimentally validated targets augmented by additional manual curation of the literature revealed 112 significantly enriched pathways from KEGG database using clusterProfiler (Supplementary Table 3 and Figures 4A–C), including the MAPK signaling (*p* = 6.18 × 10^–6^, q-value = 3.84 × 10^–5^), neurotrophin signaling (*p* = 1.66 × 10^–5^, q-value = 7.26 × 10^–5^), vascular endothelial growth factor (VEGF) signaling (*p* = 1.55 × 10^–4^, q-value = 4.22 × 10^–4^), relaxin signaling (*p* = 3.11 × 10^–4^, q-value = 6.80 × 10^–4^), and cellular senescence pathways (*p* = 6.52 × 10^–6^, q-value = 3.84 × 10^–5^). A full list of both experimentally validated targets and predicted targets from Ingenuity Knowledge Base is provided in Supplementary Table 4. ORA using GSEA additionally revealed gene sets such as neurogenesis (*p* = 3.33 × 10^–13^, FDR q-value = 1.91 × 10^–10^) and response to cytokine (*p* = 8.30 × 10^–10^, FDR q-value = 1.55 × 10^–7^; see Supplementary Table 5). Additional enriched pathways from canonical databases are provided in Supplementary Table 6 and these included the MAPK pathway from BIOCARTA,^2^ the MAPK signaling pathway from WIKI Pathways,^3^ and the MAPK signaling pathway from KEGG (48, 51) that was also identified by the clusterProfiler analysis using the same KEGG database.

## Discussion

In this study, we evaluated the relationship between circulating miRNA and the future relapse status and identified four miRNAs nominally associated with the relapse status with FDR less than 0.1. Enrichment analysis of experimentally validated targets of these four miRNAs revealed 112 statistically significant pathways enriched among the experimentally validated targets of these four miRNAs, including neurogenesis, response to cytokine, neurotrophin signaling, VEGF signaling, relaxin signaling, and cellular senescence pathways, which were implicated in MDD previously. Higher expression level of four miRNAs (*hsa-miR-199b-5p, hsa-miR-143-3p, hsa-miR-200a-3p*, and *hsa-miR-215-5p*) identified as being nominally associated with lower risk of future MDD relapse in this study. *Hsa-miR-199b-5p* was implicated in neurogenesis, MAPK signaling, and BDNF signaling pathways, all of which were statistically enriched among the experimentally validated targets of the four miRNAs identified in the current study. The *hsa-miR-199b-5p* expression level increases during early brain development (52). Compared to healthy control, *hsa-miR-199b-5p* was up-regulated in neural progenitor cells and neurons differentiated from iPSC of patients with Rett syndrome (RTT) carrying mutations in the methyl-CpG-binding protein 2 (*MECP2*) gene (52). *MECP2* has been implicated in RTT, a neurodevelopment disorder, and other neuropsychiatric conditions including depression and cocaine abuse (53). iPSC derived from *MECP2* mutants was defective in neurogenesis, and overexpression of *hsa-miR-199b-5p* resulted in a similar phenotype (52). In contrast, inhibition of *hsa-miR-199b-5p* could partially rescue the *MECP2* deficiency (52). Thus *hsa-miR-199b-5p* was placed in a neurogenesis pathway downstream of *MECP2* and its function was mediated via extracellular signal-regulated kinase (ERK/MAPK) (52). As such MAPK signaling pathway was enriched among the experimentally validated miRNA targets including mitogen-activated protein kinase 1 (*MAPK1*), mitogen-activated protein kinase 7 (*MAPK7*), mitogen-activated protein kinase 12 (*MAPK12*), mitogen-activated protein kinase kinase 4 (*MAP2K4*), protein kinase cAMP-activated catalytic subunit beta (*PRKACB*), KRAS proto-oncogene, GTPase (*KRAS*), transforming growth factor beta 2 (*TGFB2*), and Rac family small GTPase 1 (*RAC1*). *MAP2K4* is a target of *hsa-miR-200a-3p*, while *MAPK7* and *MAPK12* are targets of *hsa-miR-143-3p* according to the curations from Ingenuity Knowledge Base.

MeCP2 protein contributes to ELS-dependent epigenetic programming as phosphorylation of MeCP2 leads to hypomethylation of corticotropin releasing hormone (*CRH*), arginine vasopressin (*AVP*), and proopiomelanocortin (*POMC*, encoding a prohormone for adrenocorticotropic hormone (ACTH), a key mediator of the adrenocortical response to stress), and upregulation of these transcripts and ultimately enhancement of HPA-axis activity (54–57).

MECP2 regulates the expression of the brain-derived neurotrophic factor (*BDNF*) gene and genes modulating neuronal physiology such as calcium/calmodulin-dependent kinase *CAMK2D* and the voltage-gated potassium channel *KCNH7*, and genes involved in axon guidance and synapse formation such as *EPHA7*, *SDK1* and *CNTN4* (58–60). Mice expressing a truncated version of MeCP2 display anxiety-like phenotype and has abnormal stress response and elevated serum corticosterone levels, as the truncated form of MeCP2 fails to bind to the CpG rich promoter region of the *CRH* gene (54). In addition, mice carrying a genetic knock-in mutation that eliminates the phosphorylation site of MeCP2 also exhibit depressive-like behaviors and do not respond to chronic imipramine treatment (61). Decreased levels of MeCP2 and BDNF were detected in the hippocampus in a preclinical CUMS-induced rat model for depression and in the blood of depressed patients (62). Escitalopram improves the expression of MeCP2 in the CUMS depression model (63). Among the targets of differentially expressed miRNA, the BDNF signaling pathway was also enriched (Supplementary Table 6).

*Hsa-miR-199b-5p* is likely implicated in inflammation as it was reported to be correlated with absolute neutrophil count (64) and upregulated in tuberculosis patients (65). The immune pathways (Th1 and Th2 cell differentiation, IL-17 signaling pathway, tuberculosis, TNF signaling pathway, and T cell receptor signaling pathway in Supplementary Table 2) were also enriched in our study.

*Hsa-miR-215-5p* is another miRNA differentially expressed in the current study. It was up-regulated in the synaptosome in MDD in a human postmortem brain study where synaptic and total tissue fractions were obtained from the dorsolateral prefrontal cortex (*dlPFC*) of MDD and healthy patients (66). In our study lower expression levels of *hsa-miR-199b-5p and hsa-miR-215-5p* were associated with worse disease course (i.e., relapse in the follow-up phase).

*MiR-200a-3p*, another miRNA differentially expressed in the current study, was proposed to be a biomarker of central sensitization in chronic pain and depression. In unpredictable chronic mild stress (UCMS) and spared nerve injury (SNI) to illicit depressive-like and chronic pain behavior, *miR-200a-3p* was reduced 4 weeks after chronic stress in the prefrontal cortex (67). Pathways such as the glucocorticoid receptor (GR) signaling pathway and neurogenesis were positively regulated by *miR-200a-3p* after 4 weeks of stress, reflecting the activation of the HPA axis to overcome the stress (67). In our study, *miR-200a-3p* was downregulated in patients prone to relapse, consistent with the lower expression level when the rats exhibited depressive-like phenotype.

In rheumatoid arthritis (RA), increased expression of miR−143−3p is accompanied by promoting cell proliferation, inflammatory cytokine secretion, and inhibiting apoptosis (68), which is in contrast to a study using an Alzheimer’s disease (AD) cell model where *miR-143-3p* inhibition promotes neuronal survival (69). In the current study, a lower expression level of miR−143−3p was associated with future relapse. miR−143−3p inhibition also suppressed the Ras/p38 mitogen activated protein kinase (MAPK) signaling pathway, whereas TNF−α treatment stimulated it (68). TNF signaling pathway was also enriched in this study (Supplementary Table 2).

The study has several limitations which merit comment. Firstly, even though we report four miRNAs associated with future relapse status with a false discovery rate of less than 10%, we did not observe any miRNAs with FDR less than 5%. Secondly, the field has half a dozen studies on circulating miRNA for comparing MDD vs. healthy control, however, there is still no consensus on the definitive miRNAs associated with MDD, most likely owing to the effect size and that individual study did not have the power to unequivocally nominate the miRNA that can be replicated. Our study sample size is modest and will certainly benefit from future replication and/or meta-analysis studies examining the same endpoint. Third, the differences in miRNAs between the group who relapsed versus those that did not were based on a single time point, and the specific time course of the findings in relation to the course of depression was not established. Fourth, the differences associated with subsequent relapse have multiple possible interpretations. For example, they may reflect pathophysiological correlates which contribute to the process of impending relapse or compensatory changes in response to other factors which drove the relapse propensity.

In summary, we have identified four candidate miRNAs associated with risk of future risk and identified statistically significant pathways enriched among genes targeted by these miRNAs. Future experiments are needed to further shed light on the role and dysregulation of these candidate miRNAs in depression.

## Data availability statement

The raw data supporting the conclusions of this article will be made available by the authors, without undue reservation.

## Ethics statement

The studies involving human participants were reviewed and approved by the respective local or central Institutional Review Boards (IRBs) overseeing the clinical sites participating in the study, these included the University of Pennsylvania Office of Regulatory Affairs IRB, University of Iowa IRB, Baylor College Of Medicine IRB, University of Michigan IRB, University of Cincinnati IRB, Sharp HealthCare IRB, Springfield Committee for Research Involving Human Subjects (SCRIHS), Western IRB, University of Kansas School of Medicine – Wichita Human Subjects Committee, Rush University Medical Center IRB, Hartford Hospital IRB, University of Massachusetts Medical School IRB, Baylor College Of Medicine IRB, Stanford University Administrative Panel on Human Subjects in Medical Research, Duke University Health System IRB, Johns Hopkins Medicine IRB, IRB-III – Medical University of South Carolina, Weill Cornell Medical College IRB, and Sterling IRB. The patients/participants provided their written informed consent to participate in this study.

## Author contributions

RM, WD, and MT were involved in the conception and design of the OBSERVEMDD0001 clinical study. RM was involved in the OBSERVEMDD0001 study execution. QL was involved in the conception, design, and execution of this miRNA study and wrote the first draft of the manuscript. QL and DG were involved in the data analysis. All authors contributed to manuscript editing and revision.

## Footnotes

1
https://www.gsea-msigdb.org/gsea/msigdb/compute_overlaps.jsp

2
http://www.gsea-msigdb.org/gsea/msigdb/genesets.jsp?collection=CP:BIOCARTA

3
http://www.gsea-msigdb.org/gsea/msigdb/genesets.jsp?collection=CP:WIKIPATHWAYS
