# Supplementary material for: Circulating microRNA associated with future relapse status in major depressive disorder
Source: Front Psychiatry. 2022 Aug 17;13:937360. doi: 10.3389/fpsyt.2022.937360 (PMC9428445; doi:10.3389/fpsyt.2022.937360)

**Circulating miRNA associated with relapse status in MDD**

Qingqin S Li^1^, David Galbraith^2^, Randall Morrison^2^, Madhukar Trivedi^3^, Wayne C Drevets^4^

**Supplementary Table 1**. A list of experimentally observed targets from Ingenuity Knowledge Base + manual curation by authors

**Supplementary Table 2.** KEGG gene set enrichment results

**Supplementary Table 3.** A list of experimentally observed or predicted targets from Ingenuity Knowledge Base

**Supplementary Table 4**. ORA analysis of experimentally validated miRNA targets using GSEA resource (http://www.gsea-msigdb.org/gsea/msigdb/compute_overlaps.jsp) using C5 collection of MSigDB databases

**Supplementary Table 5**. ORA analysis of experimentally validated miRNA targets using GSEA resource (http://www.gsea-msigdb.org/gsea/msigdb/compute_overlaps.jsp) using C2 CP collection of databases

**Supplementary Figure S1** Variance partition results from the circulating miRNA dataset in this study. Genome-wide violin plot of the distribution of variance explained by each variable across all miRNAs.


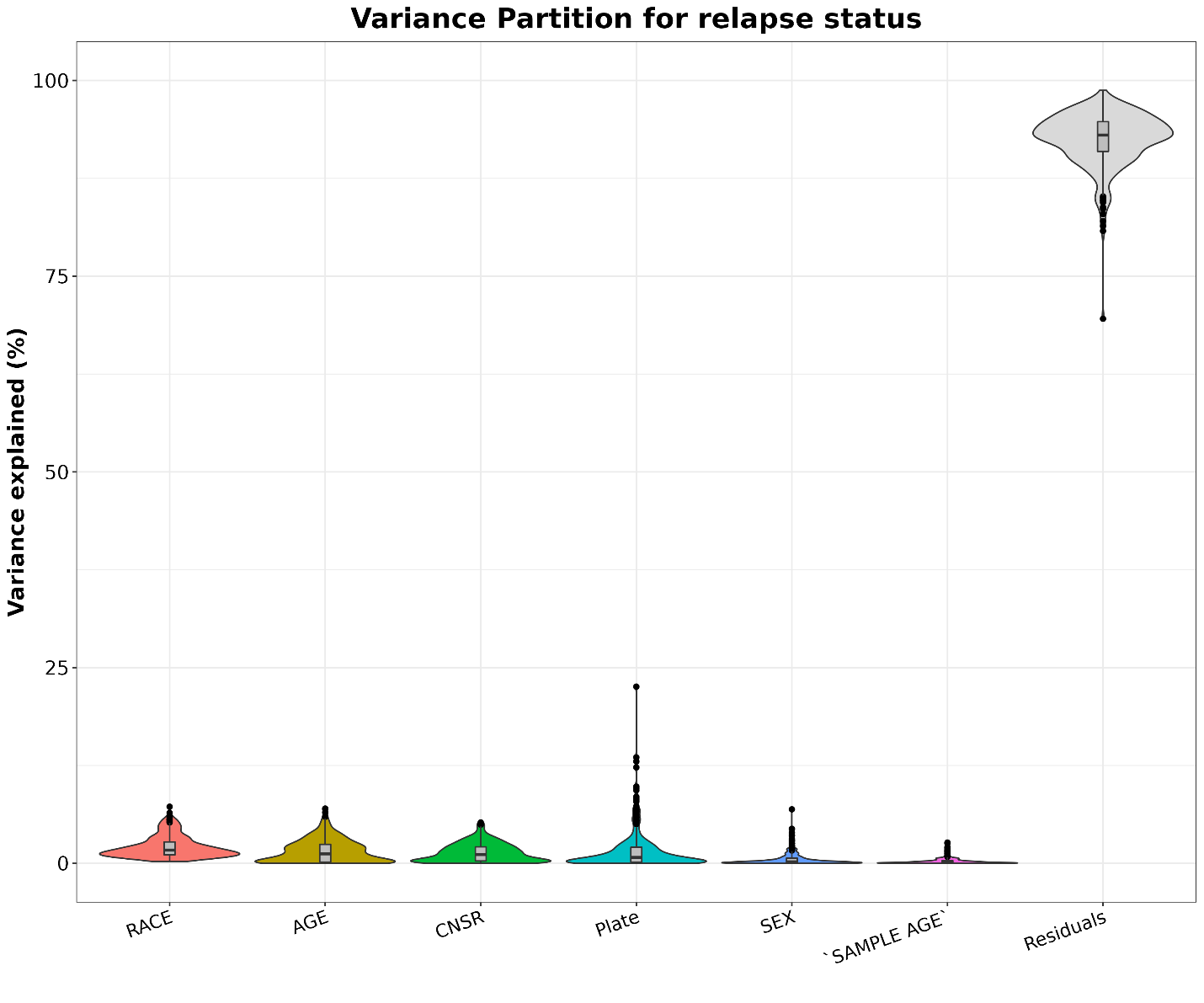


Note: The samples were assayed across two plates with roughly the same proportion between patients who relapsed vs. those who did not during follow up phase.

|  | Plate = 2 | Plate = 6 |
| --- | --- | --- |
| CNSR = 0 (relapse) | 14 | 49 |
| CNSR = 1 (not-relapse) | 35 | 119 |

**Supplementary Figure S2** Bar plots of partitioning results for the four differentially expressed miRNAs in the serum (relapse vs. non-relapse) with FDR < 0.1


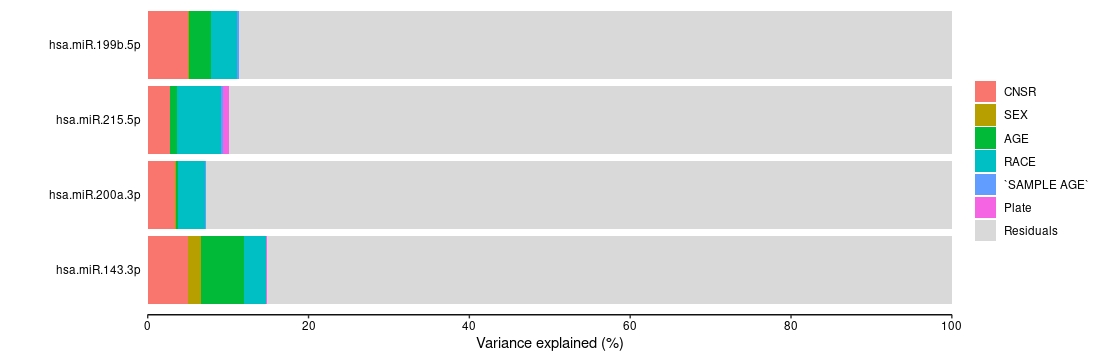


**Supplementary Figure S3** Kaplan-Meier curves for (A) *hsa-miR-199b-5p* (B) *hsa-miR-143-3p*

(A)


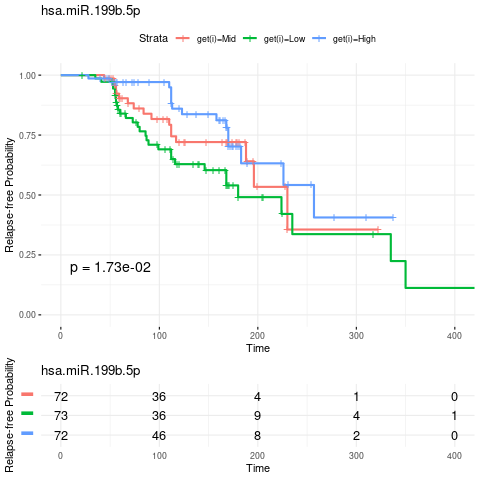


(B)


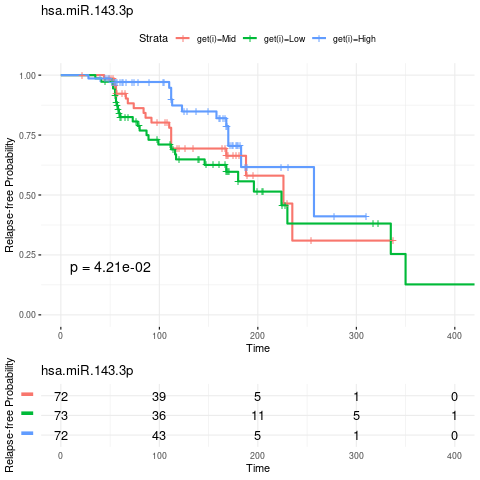

Supplement: Supplementary file 2 [file Data_Sheet_2.docx]
